# Occupational Health Programs for Artisanal and Small-Scale Gold Mining: A Systematic Review for the WHO Global Plan of Action for Workers’ Health

**DOI:** 10.5334/aogh.2592

**Published:** 2019-10-23

**Authors:** Vivian W. L. Tsang, Karen Lockhart, Samuel J. Spiegel, Annalee Yassi

**Affiliations:** 1 The World Health Organization, CH; 2 The University of British Columbia, CA; 3 The University of Edinburgh, GB

## Abstract

**Background:**

Workers in the informal economy often incur exposure to well-documented occupational health hazards. Insufficient attention has been afforded to rigorously evaluating intervention programs to reduce the risks, especially in artisanal and small-scale gold mining (ASGM).

**Objectives:**

This systematic review, conducted as part of the World Health Organization’s Global Plan of Action for Workers’ Health, sought to assess the state of knowledge on occupational health programs and interventions for the informal artisanal and small-scale gold mining (ASGM) sector, an occupation which directly employs at least 50 million people.

**Methods:**

We used a comprehensive search strategy for four well-known databases relevant to health outcomes: PubMed, Engineering Village, OVID Medline, and Web of Science, and employed the PRISMA framework for our analysis.

**Findings:**

Ten studies met the inclusion criteria of a primary study focused on assessing the impact of interventions addressing occupational health concerns in ASGM. There were no studies evaluating or even identifying comprehensive occupational health and safety *programs* for this sector although target interventions addressing specific hazards exist. Major areas of intervention—education and introduction of mercury-reducing/eliminating technology were identified, and the challenges and limitations of each intervention taken into assessment. Even for these, however, there was a lack of standardization for measuring outcome or impact let alone long-term health outcomes for miners and mining communities.

**Conclusion:**

There is an urgent need for research on comprehensive occupational health programs addressing the array of hazards faced by artisanal and small-scale miners.

## Introduction

The term informal economy was first coined by British anthropologist Keith Hart in observing the work of rural migrants in Ghana in 1971 [1]. In many low- and middle-income countries (LMICs), informal work is indeed ubiquitous, with upwards of 61% of the world’s working population make their living in the informal economy [2,3]. In 2011, according to the Pan American Health Organization (PAHO), informal work was sustaining more than 54% of the total workforce in North and South America and more than 70% in countries such as Bolivia, Honduras, and Paraguay [4]. Neither regulated, nor protected by government, activity in the informal economy generally grows in parallel with economic downturns, emphasizing that participation in the informal economy is very often out of necessity. While work-related hazards in this area are well-known, and many communities rely on it [5], only a few countries have regulations on occupational health that include the informal sector [2,4]. As access to occupational health services is a right recognized by the United Nations [6], including measures to prevent occupational risks, health surveillance, training and advice in safe working methods, and first aid, the informal sector was identified as a critical target for action in the World Health Organization (WHO) Global Plan of Action for Workers’ Health 2008–2017 as well as PAHO’s 2015–2025 plan. Consequently, at the WHO collaborating centers (CCs) meeting in Dublin 2018, CCs were asked to help synthesize the state of knowledge in this area. This review was conducted in this context, seeking to assess effectiveness of occupational health interventions implemented in a particular subset of the informal economy, namely artisanal and small-scale gold mining (ASGM).

ASGM operations are responsible for a quarter of all gold extracted globally and is often seen as a livelihood alternative for poverty-stricken communities worldwide [7,8,9]. Though at least an estimated 50 million people earn their income from ASGM [10,11,12], ASGM operations pose particularly high risks for occupational injury, exposure to mercury [13], as well as cyanide, and development of silicosis and tuberculosis amongst other diseases [14]. Environmental degradation, as a consequence of extensive excavation [15] and mercury contamination of agriculture and seafood, lead to further consequences on human health. Specifically, mercury amalgamation for gold mining continues to be a popular option due to low cost and rudimentary skill level required. This leads to increased mercury toxicity for millions of miners who are not able to access better mining technologies [16], as well as their families and communities. As such, there is great need to compile data on successful interventions not only to prevent mercury exposures, but to create effective occupational health programs for the ASGM community. Our review therefore aimed to answer the following questions:

1. What is the evidence that comprehensive occupational health programs established to address the hazards of ASGM are beneficial (or harmful)?
2. If there is no evidence regarding comprehensive programs for ASGM, what is the evidence that *any* occupational health interventions in this sector are beneficial (or harmful)?

## Methods

**Figure 1:**
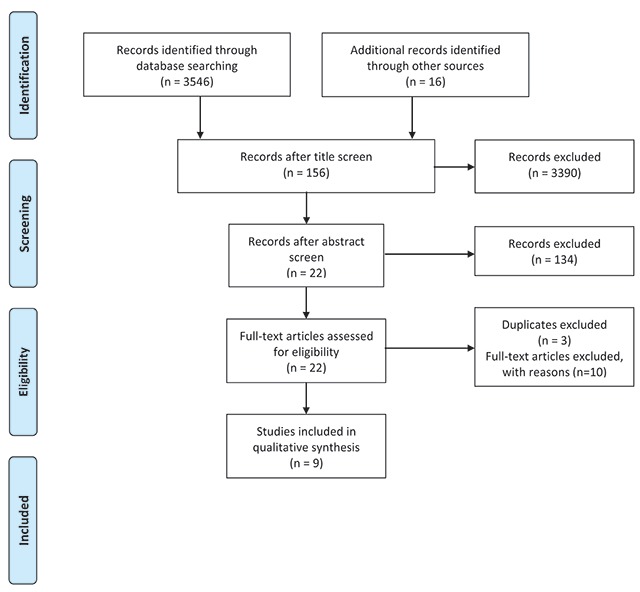
PRISMA Flow Diagram.

A broad approach was taken to gather data. Initially, a search was conducted through search engines: PubMed, OVID Medline (1946 to present), Web of Science (1900 to present), and Engineering Village which includes journals in the field of mining engineering such as Compendia, GEOBASE, and GeoRef (1884 to present) for peer-reviewed journal articles. Search terms relating to occupational health and artisanal or small-scale mining (Appendix 1) were adapted for each search engine and broadened as needed with the help of a health research librarian. The search syntax was verified with the assistance of a second librarian using WHO guidelines. Studies that were purely observational with no interventions implemented were excluded [17,18,19,20] as were those that were not primary research studies [21,22,23,24,25,26]. Studies that solely focused on large-scale gold mining were also excluded as well as those that did not focus specifically on the health of the miners themselves. Searches were restricted to articles in the English language (Figure 1, Tables 1 and 2).

**Table 1:** Inclusion criteria.


The study discusses artisanal or small-scale gold mining specifically. The study has identified health and/or safety concerns from occupational hazards or exposures. The focus of the study is on implementation of (a) possible intervention(s) to address the concerns. This is a peer-reviewed primary study, not a commentary or review. The study describes assessment of the intervention and impacts. The study is in the English language.


**Table 2:** Exclusion criteria.


The study was observational in nature and did not describe an intervention. The study did not focus on addressing occupational exposures. The study only discussed interventions on the mining community (i.e. exposures to children in mining settlements) but did not concern the miners themselves. The study only discussed environmental impacts. The study did not address human subjects. The study was not a primary research study.


One reviewer (VWLT) extracted all relevant data from eligible studies and included information such as: study properties (author names, publication year, country of study, study design and methods, sample size, type and location of intervention sites), health concerns addressed, intervention methods (design, process, materials, training), methods of evaluation (health assessments, indirect environmental impacts), and challenges for replication and expansion. This information was confirmed by another reviewer (KL) who also identified any additional studies missed on initial compilation (Tables 3 and 4). Any discrepancies or unresolved decisions for inclusion were resolved by a third reviewer (AY). Study material was contextualized by an expert and co-author with considerable experience in ASGM matters (SJS).

**Table 3:** Screening procedure.

Search Engine	Search Results	After Title Screen	After Abstract Screen	After Full-text Screen

PubMed	467	69	6	2
Engineering Village	962	35	4	2
OVID Medline	234	1	0	0
Web of Science	1883	51	12	5

**Table 4:** Results of analysis.

Study	Country	Purpose	Methods	Population	Intervention	Outcome	Limitations

Oliveira et al., 2004	Brazil	1) Evaluate methods to reduce mercury pollution and assess environmental impacts and miners’ exposures due to improved amalgamation centres 2) Assess current levels of mercury and metals in Bento Gomes river basin	Follow-up evaluation of 11 improved amalgamation centres by sampling mercury levels in air, water circuits, and precipitated solid residues after amalgamation	No direct participants	Technology: Eleven improved amalgamation centres using closed vessel systems and depositing residues from amalgamation into concrete tanks or pits sealed with plastic sheets	Emissions to local waterways reduced but inadequate reduction of mercury emissions to air Use of retorts cannot reduce mercury emissions to an acceptable level.	Initial reductions in mercury emissions can be attributed to supervision and enforcement by the FEMA as well as declining gold prices.
Drace et al., 2011	Mozambique	Assess the practices used at the Clean Tech Mine to eliminate mercury in mining processes	Survey of four ASGM sites in Mozambique, personal interviews, and site assessments of gaseous and surface soil mercury was performed.	No direct participants	Technology: Centrifugation and magnetic isolation of gold	Soil and air samples from Clean Tech mine indicate much lower mercury levels that those found at traditional ASGM sites Innovative mining practices using magnets are an inexpensive and sustainable way to eliminate mercury amalgamation practices in ASGM. These practices have strategic advantage in light of the European trade ban on mercury and United States Mercury Export Ban Act.	Limited analysis because there are no direct comparisons with other ASGM sites that have completely eliminated the use of mercury
Spiegel et al., 2013	Mozambique	1) Understand the feasibility of developing homemade retorts with local materials 2) Assess effectiveness of homemade retorts 3) Document challenges and barriers	Conducting three workshops using train-the-trainer strategies were used to introduce locally-made retorts and teach techniques to reduce mercury emissions	(At least 24 organizers from the miners’ association representing 3,764 miners in the area)	Technology: Homemade retorts; Education: Train-the-trainer three-part workshops	Portable mercury monitor noted decrease in mercury production using homemade retorts but free mercury supplies from private gold buyers are barriers to widespread adoption Recommended that government intervene to regulate gold sales and distribution	No direct numerical measurements made pre- and post-intervention to quantify the impact of retorts Long-term follow-up on success of intervention needed
Sousa and Veiga, 2009	Brazil	Capacity building and training of miners at ASGM sites focusing on the goals of: 1) Legalization of mining sites 2) Adoption of techniques to increase gold recovery 3) Protection of water and forest resources 4) Reduction of mercury use 5) Improvement in water quality, sanitation, and overall health	An initial training of trainers program found 13 people to spend 3–5 months with miners to advise them on changes in their mining operations. Balanced scorecard methodology was used to assess mining sites prior to training and evaluate success 120 days after	4,200 people at 141 mining sites	Education: Advice provided by trainers on ways to improve mining operations according to the five goals and twenty performance indicators selected	Overall conformity to indicators increased from 22.2% to 40% Reduction of mercury emissions by 10% of total mercury released in the region	Government bureaucracy and lack of resources create difficulties for miners to attain licenses to legalize mining operations. Lack of funding and resources serve as barriers in the adoption of new technologies to improve gold recovery.
Saldarriaga-Isaza et al., 2014	Colombia	Assess the feasibility and benefits of co-management and exclusion as forms of associative entrepreneurship to help mining communities invest in sustainable environmentally-friendly technologies	Multi-period two hour game hosted 1) in a classroom (n = 35) and 2) in the mines (n = 50) Participants put into small groups each with a different equipment promoted to their group Group member then decided whether they would adopt the new equipment A survey at the end of each session gathered information about personal perceptions to gold recovery process as well as assessments of risk, trust, empathy, and self-control	85 miners	Education: Adopting different institutional arrangements on associative entrepreneurship	Barriers to adoption of alternative gold mining equipment other than mercury amalgamation due to lack of funds Co-management resulted in more equal contributions and more optimal decisions and fair contribution. The leader plays a significant role in persuading other group members to make decisions for public good. Exclusion did not trigger this kind of collective action and miners made contributions that did not allow a sustained acquisition of the technology.	Base findings where total contributions exceeded the provision point was fluctuating and inconsistent leading to difficulties making comprehensive conclusions
Veiga et al., 2015	Ecuador	Reduce mercury use in ASGM practices and increase gold recovery	Miners brought to a processing plant in Portovelo Ecuador by car to demonstrate mercury-free processing techniques A classroom series was delivered by the project team - topics included explosives handling, underground ventilation, caling and falling rocks, and operational safety	211 miners from Ecuador, Colombia, and Peru	Educational workshops on methods of gravity concentration, flotation, and cyanidation to reduce mercury use	Mercury use in regions reduced by 50% in 2013 compared to levels in 2010 Thirty-nine small plants installed in Antioquia following training that used some of the practices taught Mercury levels entering processing plants reduced by 43% and mercury losses reduced 63% from comparison levels in 2010 A new training centre for artisanal miners is being built supported by the Government of Ecuador in recognition of the importance of education and training	Poor access to the mines and illegal nature of mining activities made it difficult for the research team to access trainees. Implementation of cleaner technology requires a large sum of funds
Garcia et al., 2014	Colombia	To reduce atmospheric mercury levels by focusing on improvements to the use of “entables”, retorts, and gold shops.	Use of a mercury balance to estimate mercury lost in 20 “entables” in Remedios and Segovia Absorption spectrometers were used to assess levels of atmospheric mercury. Mercury levels in urine were collected via absorption spectrometer, clinical assessments, and five neuropsychological exams conducted Introduction of new equipment and awareness campaign conducted	Health assessments performed on 50 males directly involved in mining from Segovia (mean age: 40.3) in 2010 and on 37 residents of Segovia not directly involved in mining activities in 2013	Technology: Introduction of retorts and mercury condensers, Education: Lectures for miners and owners of “entables”, awareness campaigns for mining communities, Monitoring and assessment: Atmospheric mercury and health assessments conducted	Mercury levels reduced by 63% from comparison levels in 2010 Levels of mercury in urine reduced from over 100% > 100ug Hg/g to 43% < 5 ug Hg/g, 35% > 20 ug Hg/g and 16% > 50 ug Hg/g. 39 new mercury-free processing plants generated	Bureaucratic processes and lack of understanding about project objectives and methods from stakeholders.
Metcalf and Veiga, 2011	Zimbabwe	To raise awareness about the detriments of mercury exposure using street theatre	Street theater using the *Nakai* drama performed by semi-professional travelling troupe and traditional dancers. Zimbabwe Panner’s Association hosted workshops demonstrating mercury vapour collecting retorts and gold recovery sluice carpets.	9,000 people informed about dangers of mercury exposure through the drama and 700 miners trained on safer gold recovery processes	Education: Travelling theatre	Street theater can be an effective tool for raising awareness about the hazards of mercury exposure.	Police repression on the project and corruption by nation and local elites served as barriers to proper analysis of success Actors of Nakai also did not live in mining communities themselves which contributed to lower impact. Many miners also did not stay after the performance to take part in the training workshop
Shandro et al., 2008	Mozambique	Evaluation of 9-day pilot training program in 2005 to raise awareness of impacts of mercury amalgamation and introduction to alternative practices	Visit conducted in 2007 to observe changes in ASGM practices	Not available	Technology: Centralized amalgamation centre, retorts, education, monitoring, and assessment	Use of centralized amalgamation center but improper use of retorts noted and discharge of contaminated sand Mining practices with cyanidation not yet introduced High prevalence of poverty-related diseases such as HIV and malaria noted in mining towns	Local authorities and mining associations not equipped with resources to work with researchers and miners Unsustainable due to lack of trust of trainers, lack of involvement of miners in decision-making and planning, and lack of strong awareness campaign in mining communities Short interventions neglect socioeconomic and cultural considerations

## Results

Initially 3,546 articles were identified in four different search engines (467 in PubMed, 962 in Engineering Village, 235 in OVID Medline, and 1,883 in Web of Science). The titles of these articles were screened for relevance and 3,390 articles were excluded. Abstracts of 156 articles were then screened for relevance and 23 articles remained after this process. After full text screening, 10 peer-reviewed articles were included for analysis (Table 3). References in these papers were also considered for possible inclusion.

The ten articles that met the criteria all discussed isolated interventions conducted within South America (5) and Africa (4) (Table 4) although some were part of international initiatives that extended into other continents. Of the studies included, most had short follow-ups and the initiatives themselves lacked important components of comprehensive occupational health programs. The most extensive initiative, by far, is the Global Mercury Project (GMP)—a large-scale collaboration which includes projects in Africa, South and Central America, and Asia. The GMP emphasizes environmental awareness, introduction of technology, and the use of educational campaigns in ASGM sites that primarily rely on the rudimentary process of mercury amalgamation for gold extraction. This collaboration, an initiative jointly developed in 2002 by The United Nations Environment Program, Natural Resources Defense Council, and The United Nations Industrial Development Organization, aimed for worldwide ASGM reduction in mercury pollution, seeking to build upon ASGM advancements around the world. Aside from the GMP, we found no other major international collaborations dedicated to ASGM. Here, we evaluate all occupational health interventions that met our criteria, specifically with evidence of impacts relevant for human health.

The interventions studies were summarized into two loose categories: education and technological improvements (Table 4), albeit recognizing that technological improvements are generally accompanied by at least some training. Indeed, in three of the articles [27,28,29], technological interventions were explicitly introduced with an accompanying educational component—teaching miners about ASGM-related occupational exposures and best practices of using new equipment. Some studies included measurements of both environmental *and* health impacts *as well as* educational campaigns. While pre and post intervention assessment constituted a necessary inclusion criterion in this systematic review, studies that reported monitoring results, but without describing specific hazard reduction *interventions*, were excluded.

### Technology

Six studies that discussed technological improvements introduced equipment such as homemade retorts, wet-spray misting, sluice boxes, and modified speaker magnets in lieu of mercury amalgamation [27,28,29], Three of these studies discussed single technological interventions [29,30,31]. It was found that equipment that is too expensive, or requiring complex operational procedures, was not suitable for contexts that lack supportive infrastructure or knowledge to maintain and operate such devices [30]. Examples include the regular use of Lumex spectrometers to assess mercury values, or multi-operator machinery used in larger mines. Newer methods such as gravimetry [26] are not widespread nor financially feasible for use in ASGM. No studies assessing these interventions showed short- or long-term benefits for human health.

Some studies discussed creative modifications to existing mining tools and infrastructure, including homemade retorts [27,30], mercury condensers, air filters, and activated mercury [28]. These efforts were particularly notable in the Colombia Project (CMP) targeting five municipalities in Antioquia Colombia whose levels of mercury pollution from ASGM reached the highest worldwide in 2010. New technological advances enhanced by local government enforcement contributed to an objectively successful intervention; specifically, health assessments conducted prior to and following the interventions showed reduction of mercury in urine from over 100ug Hg/g in 100% of participants tested in 2012 to <5 ug Hg/g in 43% of participants, >20 ug Hg/g in 35% of participants and >50 ug Hg/g in only 16% of participants in 2015; follow-up after three years with objective measure of health outcomes demonstrated the effectiveness of the intervention [28].

Oliveira et al.’s study evaluated interventions in the Poconé region that were in effect since the 1990s [15], including improved amalgamation centers, use of retorts, re-circulation of water in a closed system, and secure disposal of amalgamation residue. However, it was noted that despite the use of retorts, atmospheric mercury levels were still well above levels recommended by the WHO.

One local creative approach that deviates from the common use of retorts and amalgamation techniques was offered in Mozambique [29]. As opposed to mercury amalgamation, repurposed speaker magnets from old radios were used to remove iron and magnetite, leaving behind 89–93% pure gold flakes. Measured soil concentrations of mercury in the soil around the mine resulted in a finding of 0.02–0.49 mg Hg/kg of soil. These levels are much lower than those found in traditional ASGM operations such as those found in Tanzania and Shaanxi Province which had a 5- and 150-fold respective higher mercury concentration in soil comparison to the CleanTech Mine.

The only non-mercury focused study was of wet-spray misting nozzles and wet-stream machines to reduce lead and silica dust in ASGM operations, decreasing both direct respiratory and take-home exposures [31]. This was the first intervention study we could find in academic literature to focus on lead or silica exposure reduction and the first to apply water spray misting technologies in ASGM. As a result of the installation of a water tank and plumbing lines with spray mist nozzles and relocation of processing machines, Gottesfeld at al. achieved significant reductions in lead and silica dust; airborne lead and silica levels were reduced 95% and 80% respectively through wet-spray misting [31].

### Education

Educational campaigns were a clear focus in the articles that discussed interventions within the Global Mercury Project [27,30,32,33] but were also adopted in four other studies [15,16,28,34]. Education-specific studies that did not relate to technological improvements [18,35] but met our inclusion criteria sought to reduce occupational exposures through alteration of current mining practices and non-specific training and awareness of the dangers of mercury exposure.

It was reported in various studies that many traditional ASGM practices are driven by beliefs embedded within the gold-mining process; for example, in one study, dialogue with miners in Antioquia, Columbia revealed beliefs that recovering mercury used in amalgamation processes would cause a portion of gold to be lost [28]. Measuring ambient mercury levels pre- and post-education led the authors to conclude that a training-of-trainers approach where a cohort of miners are trained and then responsible for sharing learned practices within their own communities can be quite effective [34].

One study evaluated a three-day workshop in 2013 where train-the-trainer strategies were employed [35] with topics covered including the dangers of mercury exposure and environmental risks as well as how to create homemade retorts using kitchen bowls or water pipes and alternative methods of mining using a magnetic sluice box. The use of retorts also significantly lowered the amount of mercury in the air from a measurement before the intervention of 60,000 ug/m^3^ to 10ug/m^3^ after the installation of retorts, bringing levels below the recommended upper limit of 25 ug/m^3^ set by the WHO for long-term exposure to metallic mercury [36]. A previous study in 2005 [30] studied a longer 9-day training program in Munhena, Mozambique on how to create homemade retorts using kitchen bowls or water pipes to prevent inhaling mercury vapor, as well as about sodium amalgam techniques to improve recovery of gold and recycling of mercury. However, although follow-up evaluations in 2007 revealed the use of a centralized amalgamation site and regular use of retorts, improper techniques were observed which resulted in the discharge of contaminated sand. As such, no benefit was demonstrated. This work is embedded within that of the GMP.

Initial pioneers, Sousa and Veiga, performed assessments in Brazil in 2002 where extensive integration and observation of mining communities were conducted by trainers over periods of three to five months to navigate cultural and behavioural contexts which allowed them to cater suggestions to improve gold recovery. A balanced scorecard method [37] using 20 performance indicators was used to evaluate the impact of training after 120 days [33]. Although adherence to the indicators improved from 22.2% to 51%, there were minimal health assessments conducted prior to intervention and no long-term follow-up. In 2010, Veiga et al. [34] performed similar educational workshops in Ecuador to teach miners about alternative mining and processing methods using gravity concentration, flotation, and cyanidation, but again, no longer-term follow-up was reported. Saldarriaga-Isaza et al. [16] used a framed field experiment to address structural funding issues by introducing concepts of co-management and exclusion, evaluating the effectiveness of this approach through surveys to assess changes in attitudes and perceptions about the gold mining process.

## Discussion

Considerable attention has been devoted to occupational hazards in the mining sector overall [47,48,49,50,51] and much advancement has been made towards reducing risks in some areas. Yet, despite the fact that millions of miners work in artisanal and small-scale operations and make enormous contributions to world economy and poverty alleviation, research has been lacking on the effectiveness of occupational health interventions in this component of the sector. Indeed, our finding that no studies were published which evaluated comprehensive occupational health programs for ASGM is disheartening, but not surprising given the informal nature of this work. PAHO aims for 20 countries to implement comprehensive health programs in the informal sector by 2025; and WHO specifically proposes integrating basic occupational health services into primary healthcare services “especially for workers in the informal sector” (article 23). Nonetheless, the fact that ASGM activities are not only unregulated, but often illegal, is known to deter workers from accessing general health services when they suffer occupational injuries or disease [38]. Therefore, while efforts at sustainable alternative livelihoods must continue to be prioritized, targeted comprehensive occupational health programs for ASGM may indeed be warranted. This research aimed to contribute to WHO recommendations in this regard; the findings of this systematic review, however, indicate that the standard of evidence required for such recommendations is not yet attainable. As such, research on the effectiveness of interventions, and eventually of comprehensive occupationally programs for ASGM, is critical.

Uptake of mercury-free techniques has been slow, due to large sum of funds needed upfront to install machinery, buy equipment, or alter operational procedures. However, studies have documented that simple devices such as homemade retorts and education about proper mercury disposal can effectively reduce mercury exposure, and educational interventions such as workshops and training sessions that supplement the introduction of new mining equipment can help. The use of magnets in lieu of mercury amalgamation is reasonably scalable, and efforts can be put into sharing best practices with other mining operations. However, systematic documentation and evaluation of long-term impacts [39] has been lacking. As the Minamata Convention on Mercury adopted in 2013 includes actionable guidelines to phase out mercury use in ASGM, long-term health assessments are also needed in conjunction with this Treaty.

Saldarriaga-Isaza et al. [16] stress the importance of collective action and other intra- or intercommunity partnerships in implementing health protection measures for ASGM. Additionally, a systematic understanding of the social metabolism of mercury is warranted, in which local knowledge is combined with an analysis of national and international power relations [12] to determine what is feasible. Too often, funding allocated for education-related interventions are bound by donor specifications, [40] inadequately contextualized in each mining community, [30] laws and regulations of different regions, as well as cultural and power dynamics, economics, and geology. [40,41] Access to land and licensure required in different areas further act as barriers in uptake of technological innovations [12].

A framework for training miners in ASGM can be employed to improve efficacy and streamline education interventions for miners [40], for example recruiting trainers who are prominent community leaders in positions of influence [40]. With greater collaborations, standards of reporting occupational health outcomes can also be regulated so that future intervention studies can be easily compared. Increased adoption of suggestions that disrupt long-held mining practices can be reinforced by local government and mining associations further incentivizing uptake of alternatives [42]. Governments in many countries are only offering official assistance to people working in licensed or registered small-scale mining zones, marginalizing those operating in informal mining arrangements [12,40]. In particular, women face the brunt of many negative impacts [43] as miners in ASGM and also in processing or gold decomposition [44], leading to increased exposures to mercury and injury [45]. Within mining communities, sexual abuse and violence against women has been reported with high rates of morbidity and mortality due to untreated sexually transmitted diseases [45]. However, women also utilize ASGM as a unique means of income to achieve social and financial independence [46] and indeed have been able to take advantage of programs such as microfinancing [44]. In planning occupational health services, the special needs of women need to be considered. Addressing relational and structural inequities that limit the uptake of education and technological innovations will be key in establishing needed occupational health services.

Lastly, our finding of only one study of interventions reducing exposures to hazards *other* than mercury [31] underlines the need for research addressing a variety of ASGM-related hazards [13].

### Limitations

The databases used in this study focus on English language articles. However, no studies were found in a non-English language using the aforementioned broader search strategy.

## Conclusion

Few peer-reviewed studies exist that evaluate occupational health interventions for this population. This is not because of the lack of documentation of the hazards in this sector. The establishment of treaties like the Minamata Convention on Mercury coupled with new innovations in technology will undoubtedly help reduce mercury use, as will promote other preventive measures in ASGM. However, the lack of documentation of the effectiveness of interventions is problematic. Whether the dearth of literature reflects lack of occupational health interventions and services, or rather absence of studies thereof, cannot be determined by our review.

Moreover, even beyond exposures to mercury, lead, mercury and other toxic substances, occupational hazards in ASGM include concerns about hygiene and sanitation, and the rise of sex workers and associated reproductive health problems in mining communities. Whether interventions exist to address these concerns exist but have not been evaluated, or rather have not been implemented at all, cannot be determined by this study. What we can conclude is that there remains a lack of meticulous pre- and post-intervention health assessments to facilitate the objective measurement of success in improving health and wellbeing for this sector. While efforts to find alternate livelihoods for those working in precarious and hazardous situations is imperative, research is urgently needed now as to how to offer occupational health interventions, if not full programs, to mitigate the risks.

## Additional File

The additional file for this article can be found as follows:

10.5334/aogh.2592.s1 Appendix 1. Search Criteria.
