# Supplementary material for: Occupational Health Programs for Artisanal and Small-Scale Gold Mining: A Systematic Review for the WHO Global Plan of Action for Workers’ Health
Source: Ann Glob Health. 2019 Oct 23;85(1):128. doi: 10.5334/aogh.2592 (PMC6813427; doi:10.5334/aogh.2592)
Supplement: Appendix 1. — Search Criteria. [file agh-85-1-2592-s1.pdf]

Appendix Search Criteria:

(occupational [tw] OR worker*[tw] OR employe*[TW]) OR "Occupational Health"[Mesh] OR "Occupational Health Physicians"[Mesh] OR "Occupational Health Services"[Mesh] OR "Occupational Health Nursing"[Mesh] OR “industrial hygiene” [TW] OR “occupational safety” OR “employee health” [TW] OR “occupational health” [TW] OR “company physician” [TW] OR “company physicians” [TW] OR “Employee Health Service” [TW] OR “Employee Health Services” [TW] OR “Employment-Based Service” [TW] OR “Employment-Based Services” [TW] OR “industrial nursing” [TW] OR “company nurse” [TW] OR “company nurses” [TW])) AND (((((informal sector) OR informal economy) OR small scale mining) OR artisanal mining) OR small-scale mining) OR (illegal mining))))))

Returns about 467 results on PubMed

After title screen - 69 remaining

After abstract screen - 6 remaining

After full text screen - 2 remaining

(((((((((((((((((((((((artisanal NEAR mining) WN KY) OR ((small$scale NEAR mining) WN KY)) OR ((informal economy) WN KY)) OR ((informal sector) WN KY)) OR ((illegal NEAR mining) WN KY)) AND ((occupation*) WN KY)) OR ((worker*) WN KY)) OR (("Occupational Health") WN KY)) OR (("Occupational Health Physician*”) WN KY)) OR (("Occupational Health Service*”) WN KY)) OR (("industrial hygiene") WN KY)) OR (("occupation* safety") WN KY)) OR (("employ* health") WN KY)) OR (("occupation*

health") WN KY)) OR (("company physician*") WN KY)) OR ((“Employee Health Service*”) WN KY)) OR (("Employment$based Service*") WN KY)) OR (("industrial nurs*”) WN KY)) OR (("company nurs*") WN KY)) OR ((exposure) WN KY)) OR ((industrial) WN KY)) OR ((environment* NEAR health) WN KY))

Returns 13575732 on Engineering Village
After duplicates removed and Compendex as chosen database 962 total (819 compendia, 133 GEOBASE, 10 GeoRef)
After title screen - for information re: small scale mining or artisanal mining with OHS activities of any sort or those about mining or interventions and assessments on informal sector that may have mining as one of the evaluations- 35 selected
After abstract screen - for information re intervention on ASM activities of any sort - 4 remaining
After full text screen - 2 remaining or 4 if you include the reviews and not just independent studies

(artisanal NEAR mining or small$scale NEAR mining or informal economy or informal sector or illegal NEAR mining).mp. [mp=title, abstract, original title, name of substance word, subject heading word, floating sub-heading word, keyword heading word, protocol supplementary concept word, rare disease supplementary concept word, unique identifier, synonyms]

(occupation* or worker* or "Occupational Health" or "Occupational Health Physician*" or "Occupational Health Service*.af. OR industrial hygiene .af. OR occupation* safety .af. OR

employ* health .af. OR occupation* health .af. OR company physician* .af. OR Employee Health Service* .af. OR Employee$based Service* .af. OR industrial nurs* .af. OR industrial.af. OR exposure.af. OR company nurse*" or environment* NEAR health).mp. [mp=title, abstract, original title, name of substance word, subject heading word, floating sub-heading word, keyword heading word, protocol supplementary concept word, rare disease supplementary concept word, unique identifier, synonyms]

Returns 234 on OVID MEDLINE

After title screen - 1

After abstract screen - 0

(TS=(occupation*) OR TI=(occupation*) OR TS=(worker*) OR TI=(worker*) OR TS=(“Occupational Health") OR TI=("Occupational Health") OR TS=("Occupational Health Physician*) OR TI=("Occupational Health Physician*) OR TS=(”Occupational Health Service*") OR TI=(”Occupational Health Service*")OR TS=("industrial hygiene") OR TI=("industrial hygiene") OR TS=("occupation* safety") OR TI=("occupation* safety") OR TS=("employ* health") OR TI=("employ* health") OR TS=("occupation* health") OR TI=("occupation* health") OR TS=("company physician*") OR TI=("company physician*") OR TS=(Employee Health Service*) OR TI=(Employee Health Service*) OR TS=(Employee%Based Service*) OR TI=(Employee%Based Service*) OR TS=("industrial nurs*") OR TI=("industrial nurs*") OR TS=("company nurs*") OR TI=("company nurs*") OR TS=(exposure) OR TI=(exposure) OR TS=(industrial) OR TI=(industrial) OR TS=(environment* NEAR health) OR TI=(environment* NEAR health))

AND

(TS=(artisanal NEAR mining) OR TI=(artisanal NEAR mining) OR TS=(small$scale NEAR mining) OR TI=(small$scale NEAR mining) OR TS=(illegal NEAR mining) OR TI=(illegal NEAR mining) OR TS=(informal economy) OR TI=(informal economy) OR TS=(informal sector) OR TI=(informal sector))

Returns 1883 on Web of Science

After Title Screen - also removed anything non-human or not ASM specific or ASM but about something else i.e. sex and culture - 51

After abstract screen - 12

After full text screen - 6 or 7 if UN development paper is found
